# Plasmid-Mediated Colistin Resistance (*mcr-1*) in *Escherichia coli* from Non-Imported Fresh Vegetables for Human Consumption in Portugal

**DOI:** 10.3390/microorganisms8030429

**Published:** 2020-03-18

**Authors:** Vera Manageiro, Daniela Jones-Dias, Eugénia Ferreira, Manuela Caniça

**Affiliations:** 1 National Reference Laboratory of Antibiotic Resistances and Healthcare Associated Infections (NRL-AMR-HAI), Department of Infectious Diseases, National Institute of Health Dr. Ricardo Jorge, 1649-016 Lisbon, Portugal; vera.manageiro@insa.min-saude.pt (V.M.); d.jones.dias@gmail.com (D.J.-D.); eugenia.ferreira@insa.min-saude.pt (E.F.); 2 Centre for the Studies of Animal Science, Institute of Agrarian and Agri-Food Sciences and Technologies, Oporto University, 4051-401 Oporto, Portugal

**Keywords:** *mcr-1*, colistin resistance, food, vegetables

## Abstract

In this study, we report the presence of the plasmid-mediated colistin resistance (PMCR)-encoding gene *mcr-1* in an *Escherichia* *coli* isolate, INSali25, recovered from lettuce produced and marketed in Portugal. Colistin MIC from the vegetable *E. coli* isolate—determined by microdilution broth method according to EUCAST guidelines—revealed a non-wild-type phenotype of colistin (MIC 16 mg/L). To understand the genetic background of *E. coli* INSali25, we performed whole genome sequencing. Plasmid sequencing was also performed after plasmid DNA extraction from the transconjugant TcINSali25 (*mcr-1*). Directed bioinformatics analysis identified the *mcr-1* gene in a 39,998 bp length contig, with an upstream region including the antibiotic resistance gene *bla*_TEM-1_ in a partial transposon Tn*2*, truncated by the insertion sequence IS*26* and showing >99% identity with previously described *mcr-1*-harboring IncHI2 plasmids. Further in silico analysis showed the presence of additional genes conferring resistance to β-lactams (*bla*_TEM-1_), aminoglycosides (*aadA1*, *aph(4)-Ia*, *aph(6)-Id*, *aac(3)-Iv*), macrolides (*mdf(A)-*type), phenicol (*floR-*type), tetracycline (*tetA*), and sulphonamides (*sul2*). INSali25 isolate belonged to the ST1716 lineage and showed the *fimH54* and *fumC27* alleles. Lettuce is a vegetable that is commonly consumed fresh and not subjected to any cooking process, which may amplify human food safety risks. Moreover, the occurrence of plasmid-mediated colistin resistance in a sample that was not imported and was acquired in a large retail store reinforces the widespread distribution of *mcr-1*.

## 1. Introduction

Colistin constitutes one of the few therapeutic options available for the treatment of infectious diseases caused by multidrug resistant Gram-negative bacteria [1]. However, plasmid-mediated colistin resistance (PMCR) determinants have been detected in humans and animals, and intra-species transmission of resistant isolates has already been reported [2,3]. Fresh produce has been increasingly implicated in bacterial outbreaks. Indeed, fruits and vegetables can become contaminated with antibiotic resistance bacteria before wholesale distribution [4]. Until now, three studies reported MCR-1-producing *Enterobacteriaceae* isolated from fresh produce, all originated from Asia. In 2016, four *E. coli* and two *Raoultella ornithinolytica* recovered from lettuce and tomato retail vegetables was reported in China [5]; other study described two *E. coli* isolated in Switzerland from ready-to-eat vegetables imported in 2014 from Thailand and Vietnam [6]. Recently, twenty-three MCR-1-producing *E. coli* and one MCR-1-producing *Enterobacter cloacae* were isolated from fresh vegetable samples collected between May 2017 and April 2018, in nine provinces in China [7].

Therefore, the aim of this study was to characterize a PMCR-encoding gene *mcr-1* detected in an *E. coli* isolated from a lettuce sample in Portugal and evaluate its genetic relation with the other reported MCR-1-producing *E. coli* isolated from fresh produce.

## 2. Materials and Methods

### 2.1. Bacterial Isolate

In the scope of the analysis of the antibiotic nonsusceptibility of a collection of Gram-negative isolates recovered from fresh fruits and vegetables, we identified the presence of the PMCR-encoding gene *mcr-1* in *E. coli* INSali25 isolate, which was recovered from lettuce acquired at a retail store [8]. This lettuce, produced in conventional agriculture and marketed in Portugal, was purchased at a retail store in the region of Lisbon and Tagus Valley [8].

### 2.2. Antimicrobial Susceptibility Testing

Minimum inhibitory concentrations (MICs) were determined by microdilution methods as previously described [9] and interpreted according to European Committee of Antimicrobial Susceptibility Testing (EUCAST, http://www.eucast.org/).

### 2.3. Transfer Experiments

Conjugation experiments were performed using sodium azide-resistant *E. coli* J53 as a recipient strain [9]. Transconjugants were selected on McConkey agar supplemented with sodium azide (150 mg/L) and colistin (2 mg/L).

### 2.4. Genetic Background of E. coli INSali25

To understand the genetic background of *E. coli* INSali25, we performed whole genome sequencing (WGS) (MiSeq, IlluminaInc, San Diego, CA, USA) and bioinformatics analyses, as previously described [8]. This Whole Genome Shotgun (WGS) project was deposited at DDBJ/ENA/GenBank under the accession LSRK00000000. The version described in this paper is version LSRK02000000.

Plasmid sequencing was also performed on a MiSeq Illumina platform using 150 bp paired-end reads, after plasmid DNA extraction from TcINSali25 (*mcr-1*) using a NucleoBond Xtra Plus kit (Macherey-Nagel, Dueren, Germany). Plasmid sequence reads were trimmed and filtered according to quality criteria and were mapped back to the contigs generated by WGS—which were used as references by means of CLC Genomics Workbench 10.0 (Qiagen, Aarhus, Denmark). Briefly, the raw FASTQ reads were first processed by quality score trimming (quality score limit  =  0.05), removing all reads containing more than 2 ambiguous nucleotides or shorter than 50 bp. Trimmed reads were then de novo assembled with automatic bubble, word size and paired distance detection, using mapping mode, “map reads back to contigs” (including scaffolding and minimum contig length of 400 nucleotides).

Twenty contigs (coverage ≥ 10× and length ≥ 500 bp) were obtained, which were BLAST searched against the Microbial Nucleotide BLAST database for complete plasmids. By mapping against plasmid hits, we were able to obtain a larger contig containing the *mcr-1* gene (with 39,988 bp, including contigs 106, 119 and 136). Contigs were analyzed, molecular typed, and studied for the presence of antibiotic resistance and plasmid replicon types, using publicly available Web tools hosted by the Center for Genomic Epidemiology (CGE, https://cge.cbs.dtu.dk/services/).

### 2.5. Genomic Epidemiological Analysis

The BacWGSTdb database was used for genotyping, source tracking bacterial pathogens, and prediction of closely related plasmids [10].

## 3. Results and Discussion

Colistin MIC of the vegetable *E. coli* isolate revealed a non-wild-type phenotype to colistin (MIC 16 mg/L). This isolate was also resistant to other antibiotic classes, such as penicillins, quinolones, aminoglycosides, and phenicols, consistent with a multidrug resistant phenotype. The transferability of the *mcr-1* gene was achieved, with the transconjugant TcINSali25 (*mcr-1*) exhibiting the respective resistance to colistin (4 mg/L).

*In silico* analysis revealed the presence of additional genes conferring resistance to β-lactams (*bla*_TEM-1_), aminoglycosides (*aadA1*, *aph(4)-Ia*, *aph(6)-Id*, *aac(3)-Iv*), macrolides (*mdf(A)-*type), phenicol (*floR-*type), tetracycline (*tetA*), and sulphonamides (*sul2*). Furthermore, we found a chromosomal point mutation in the *gyrA* gene (S83L) which is responsible for resistance to fluoroquinolones. One virulence factor (*gap*-type) was identified. Moreover, this *E. coli* isolate displayed a prediction of 93.4% for being a human pathogen, based on the probability scores assigned by PathogenFinder. The MCR-1-producing INSali25 *E. coli* isolate belonged to the ST1716 lineage and showed the *fimH54* and *fumC27* alleles using CHTyper. This ST was encountered worldwide mainly in isolates collected from livestock samples (http://enterobase.warwick.ac.uk/species/ecoli/).

**Figure 1:**
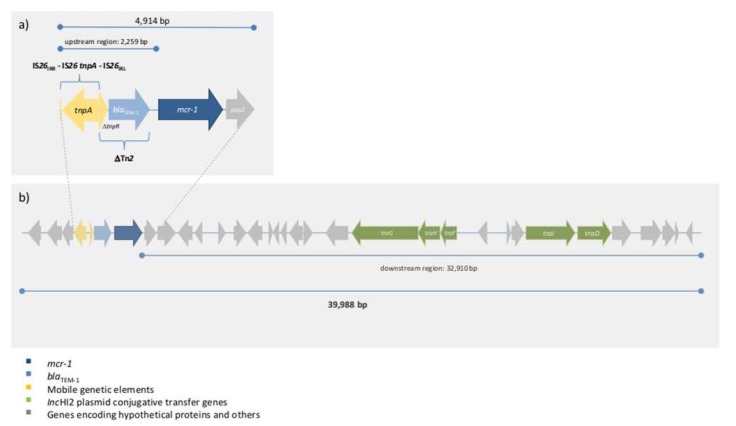
Schematic representation of *mcr-1* gene flanking regions (**a**) *mcr-pap2* element and upstream region including the antibiotic resistance gene *bla*_TEM-1_. (**b**) contig region enclosing *mcr-1* gene.

Further bioinformatics analysis directed to the flanking regions of the *mcr-1* gene identified, upstream of the *mcr-pap2* element, a 2259 bp fragment including the antibiotic resistance gene *bla*_TEM-1_ in a partial transposon Tn*2* truncated by the insertion sequence IS*26* (Figure 1a). The downstream region (32,910 bp) included multiple *orfs*, among which we highlight the presence of genes encoding plasmid conjugative transfer proteins (Figure 1b). Indeed, in silico plasmid detection and typing analyses revealed the presence of the incompatibility group HI2 (IncHI2) ST4, which has been associated with a wide dissemination of *mcr-1* gene [11].

Alignment of pINSali25-MCR contig to complete plasmids from Microbial Nucleotide BLAST database showed >99.6% identity with previously described *mcr-1*-harboring IncHI2 plasmids (Table 1). Indeed, the top six hits included IncHI2-type *mcr-1*-harboring plasmids found in isolates collected from animals, humans, and sewage in various countries. Remarkably, p1rc4-mcr1 (NZ_CM008266) from an *E. coli* isolated from the stool of a Hajj pilgrim returning from a pilgrimage from Saudi Arabia to France [12], showed >99.99% similarity to pINSali25-MCR, highlighting the importance of travelers in the spread of multidrug-resistant bacteria. However, no genetic relation was found between *E. coli* INSali25 and other reported MCR-1-producing *E. coli* isolated from fresh produce since all belonged to different MLST and/or plasmid types (Table 2). Furthermore, no *E. coli* INSali25 closely related isolates were detected among those currently deposited in the public database BacWGSTdb [10].

Despite previous reports of food products from animal origin, lettuce is a vegetable that is commonly consumed fresh and not subjected to any cooking process, which severely amplifies the human food safety risks involved [13]. Considering that colistin is a last-resource antibiotic used for the treatment of infections caused by multidrug resistant bacteria, the detection of a mobile colistin resistance gene in a raw vegetable constitutes a serious and unprecedented public health concern [1]. Indeed, the occurrence of plasmid-mediated colistin resistance in a sample that was produced in Portugal and that was acquired in a large retail store reinforces the widespread distribution of *mcr-1*, and the need to promote a global and concerted strategy to contain the spread of this resistance mechanism.

## Figures and Tables

**Table 1:** Comparison of *mcr-1.1*-harboring INSali25 contig with the top six IncHI2-type *mcr-1*-harboring plasmids showing the highest identities (>99.6%, *E*-value 0.0, query coverage >94.0%).

IncHI2-Type Plasmid (bp)	Strain (MLST ^1^)	Isolation Source/ Country/Year	Identity(%)	Alignment Length (bp)	Antimicrobial Resistance Gene ^2^	Plasmid GenBank Acc. No.
**pSE08-00436-1** (264914)	*S. enterica* 08-00436 (ST28)	Chicken skin/Germany/2008	99.997	32115	*aadA1*, *aadA2-type*, *aph(3’)-Ia-type*, *aph(3’’)-Ib-type*, *aph(6)-Id*, *aac(3)-IIa*, *bla*_TEM-1B_, *mcr-1.1*, *cml-type*, *catA1-type*, *sul1*, *sul2*, *sul3*, *tet(A)*, *dfrA1-type*	NZ_CP020493.1
**p1rc4-mcr1** (239098)	*E. coli* 1RC4 (ST155)	Hajj pilgrim Stool/France/2014	99.994	32115	*aadA1*, *aadA12-type*, *aph(3’)-Ia*, *aph(3’’)-Ib*, *aph(6)-Id*, *bla*_TEM-1B_, *mcr-1.1*, *mph(A)*, *cml-type*, *floR-type*, *sul3-type*, *tet(A)*, *dfrA4*	NZ_CM008266.1
**pSA186_MCR1** (241600)	*E. coli* SA186 (ST131)	Human patient urine/ Saudi Arabia (Riyadh)/2012	99.993	30522	*aadA1*, *aadA2-type*, *aph(3’)-Ia*, *aph(3’’)-Ib*, *aph(6)-Id*, *bla*_TEM-1B_, *mcr-1.1*, *mph(A)*, *cml-type*, *floR-type*, *sul3-type*, *tet(A)*, *dfrA14*	NZ_CP022735.1
**pRS571-MCR-1.1** (257270)	*E. coli* RS571 (ST648)	rectal swab/Bangladesh: Dhaka/2018	99.993	29648	*aadA1*, *aadA2-type*, *aac(3)-IId-type*, *aph(3’)-Ia*, *aph(3’’)-Ib*, *aph(6)-Id*, *bla*_TEM-1B_, *mcr-1.1*, *mph(A)*, *cml-type*, *floR-type*, *sul3-type*, *tet(A)*, *dfrA14*	NZ_CP034390.1
**pG3X16-2-2** (265575)	*E. coli* G3X16-2 (ST1196)	Human feces/China: Guangxi/2017	99.977	30043	*aadA22*, *aph(3’)-Ia-type*, *aph(3’’)-Ib*, *aph(6)-Id*, *aph(4)-Ia-type*, *aac(3)-IV*, *bla*_TEM-1B_, *bla*_CTX-M-65_, *mcr-1.1*, *oqxAB-type*, *Inu(F)-type*, *mph(A)*, *floR-type*, *sul1-type*, *sul2*, *tet(A)-type*, *tet(M)-type*	NZ_CP038139.1
**pMCR1_025943** (265538)	*E. coli* WCHEC025943 (ST410)	Sewage/China: Sichuan, Chengdu/2017	99.977	30043	*aadA22*, *aph(3’)-Ia-type*, *aph(3’’)-Ib*, *aph(6)-Id*, *aph(4)-Ia-type*, *aac(3)-IV*, *bla*_TEM-1B_, *bla*_CTX-M-65_, *mcr-1.1*, *oqxAB-type*, *Inu(F)-type*, *mph(A)*, *floR-type*, *sul1*, *sul2*, *tet(A)*, *tet(M)-type*	NZ_CP027202.2

^1^ MLST accordingly with Warwick scheme (http://mlst.warwick.ac.uk/mlst/dbs/Ecoli); ^2^ ResFinder-3.1 (Selected % ID threshold: 90%; Selected minimum length: 60%).

**Table 2:** Comparison of MCR-1-producing *E. coli* strains isolated from fresh produce.

*E. coli* Strain (MLST ^a^)	Isolation Source/Country/Year	Antimicrobial Resistance Genes ^b^	Plasmid (bp)	Plasmid Type	Accession Number	Reference
**INSali25** (ST1716)	Lettuce/Portugal/2015	*aadA1*, *aac(3)-Iv*, *aph(4)-Ia,aph(6)-Ia*, *aph(6)-Id, bla_TEM-1B_, mcr-1.1*, *sul2*, *tet(A)*, *floR-type*	pINSali25-MCR (≈250000)	IncHI2/ST4	LSRK00000000	[8]; This study
**CTX148** (untypable)	tomato	*mcr-1.1*	pT-CTX148 (57764)	IncI2	MK754161	[7]
**TO89** (ST713)	Tomato/China: Huimin/2017	*mcr-1.1*	pT-89 (∼33)	IncX4	SRMK00000000	[7]
**SQB-1-1** (ST2705)	Romaine lettuce/China: Qingdao/2017	*aph(3’)-Ia, aac(3)-IV-type, aph(4)-Ia, aadA1, aadA2-type, bla*_CTX-M-14_, *mcr-1.1*, *fosA3*, *mdf(A)-type*, *mph(A), cmlA1-type*, *floR-type*, *tet(M)-type*, *sul2*, *sul3*	pSQB-1-1 (≈250000)	IncHI2/ST3	SRML00000000	[7]
**H226B** (ST167)	Cha-om imported from Thailand/2014	*mcr-1.1*	pH226B (209401)	IncHI1	KX129784	[6,14]
**2SK1** (ST4683)	Basil leaves imported from Vietnam/2014	*mcr-1*, *bla*_CTX-M-65_	-	-	-	[14]
**HS20eCTX** (ST795)	Lettuce/China: Guangzhou/2016	*aph(3’)-Ia, aac(3)-IV-type, aph(4)-Ia, aadA1, aadA2-type, bla*_CTX-M-14_, *mcr-1.1, fosA3*, *cmlA1-type, floR-type, sul2, sul3*	pHNHS20EC (250827)	IncHI2/ST3	MF135536	[5]
**BS21Ectx** (ST2505)	Lettuce/China: Guangzhou/2016	*mcr-1*	- (∼60)	IncI2	-	[5]
**6BF21eCTX** (ST69)	Tomato/China: Guangzhou/2016	*mcr-1, bla*_CTX-M-14_ *, floR, fosA3, oqxAB*	- (∼244)	IncHI2/ST3	-	[5]
**TS62CTX** (ST156)	Lettuce/China: Guangzhou/2015	*mcr-1*	- (∼60)	IncI2	-	[5]
**6HS20E** (ST48)	Lettuce/China: Guangzhou/2016	*mcr-1*	- (∼33)	IncX4	-	[5]

^a^ MLST accordingly with Warwick scheme (http://mlst.warwick.ac.uk/mlst/dbs/Ecoli); ^b^ Results from ResFinder-3.1 (Selected %ID threshold: 90%; Selected minimum length: 60%) using available plasmid accession numbers; otherwise, results are those from references. - Data not available.
